# Prediction of Pre-Loading Relaxation of Bolt Structure of Complex Equipment under Tangential Cyclic Load

**DOI:** 10.3390/s24113306

**Published:** 2024-05-22

**Authors:** Xiaohan Lu, Min Zhu, Chao Li, Shengnan Li, Shengao Wang, Ziwei Li

**Affiliations:** 1 College of Nuclear Science and Technology, Naval University of Engineering, Wuhan 430033, China; 2 College of Power Engineering, Naval University of Engineering, Wuhan 430033, China

**Keywords:** pre-loading, M8 bolt, nickel steel flat plate, tangential force, performance prediction

## Abstract

Bolts have the advantages of simple installation and easy removal. They are widely applied in aerospace and high-speed railway traffic. However, the loosening of bolts under mixed loads can lead to nonlinear decreases in pre-loading. This affects the safety performance of the structure and may lead to catastrophic consequences. Existing techniques cannot be used to monitor the bolt performance status in time. This has caused significant problems with the safety and reliability of equipment. In order to study the relaxation law of bolt pre-loading, this paper carries out an experimental analysis for 8.8-grade hexagonal bolts and calibrates the torque coefficient. We also studied different loading waveforms, nickel steel plate surface roughnesses, tangential displacement frequencies, four different strengths and bolt head contact areas of the bolt, the initial pre-loading, and the effects of tangential cyclic displacement on pre-loading relaxation. This was done in order to accurately predict the degree of bolt pre-loading loosening under external loads. The laws are described using the allometric model function and the nine-stage polynomial function. The least squares method is used to identify the parameters in the function. The results show that bolts with a smooth surface of the connected structure nickel steel flat plate, high-frequency working conditions, half-sine wave, and a high-strength have better anti-loosening properties. Taking 5–10 cycles of cyclic loading as a boundary, the pre-loading relaxation is divided into two stages. The first stage is a stage of rapid decrease in bolt pre-loading, and the second stage is the slow decrease process. The performance prediction study shows that the allometric model function is the worst fitted, at 71.7% for the small displacement condition. Other than that, the allometric model function and the nine-stage polynomial function can predict more than 85.5% and 90.4%, which require the use of least squares to identify two and ten unknown parameters, respectively. The complexity of the two is different, but both can by better indicators than the pre-loading relaxation law under specific conditions. It helps to improve the monitoring of bolt loosening and the system use cycle, and it can provide theoretical support for complex equipment working for a long time.

## 1. Introduction

There are a large number of bolt structures in large and complex equipment [1,2], and the operational life of the bolt greatly affects the safety and complexity of the equipment. The bolt structure prevents the connecting structure from sticking and slipping after being subjected to a tangential load in the form of pre-loading. A small pre-loading is likely to cause the bolt to loosen, leading to safety problems in large and complex equipment and affecting the overall safety of the reactor. Excessive pre-loading can easily lead to losses in bolt strength and stiffness [3].

Pre-loading directly affects the reliability of the bolted structure of complex equipment. The magnitude of pre-loading is affected by factors such as the bolt material, diameter, bearing speed, lubricant temperature, and friction coefficient [4,5]. Yang [6] compared the loosening degree of different bolts under a tangential force, and the performance of the anti-loosening bolts was 9.43% lower than that of ordinary bolts. Hu [7] studied the effects of the bolt diameter, pitch, and friction coefficient on the size of pre-loading based on a finite element analysis based on the secondary development using Python and ABAQUS. The results showed that at the same torque, the size of the bolt diameter is inversely proportional to the pre-loading; the pitch has a small effect on the pre-loading; and the friction coefficient has a significant effect. Reference [8] points out that at lower speeds, the bolt pre-loading needs to be increased to maintain stability. In the case of mechanical finishing, the bolt pre-loading should be reduced to ensure that the stiffness is within a reasonable range. Under the coupling of multiple influencing factors, different tangential cyclic displacements cause irregular pre-loading relaxation. As a result, it is difficult to accurately evaluate the overall performance of large and complex equipment.

**Figure 1:**
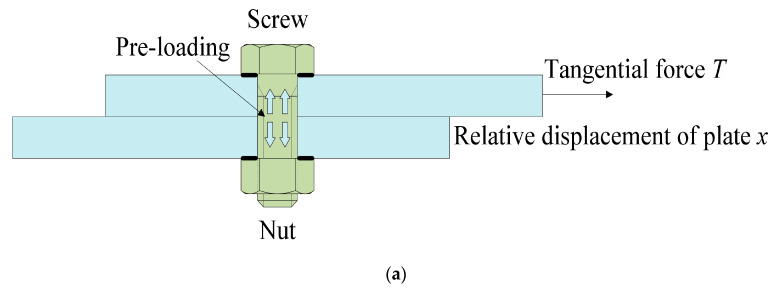

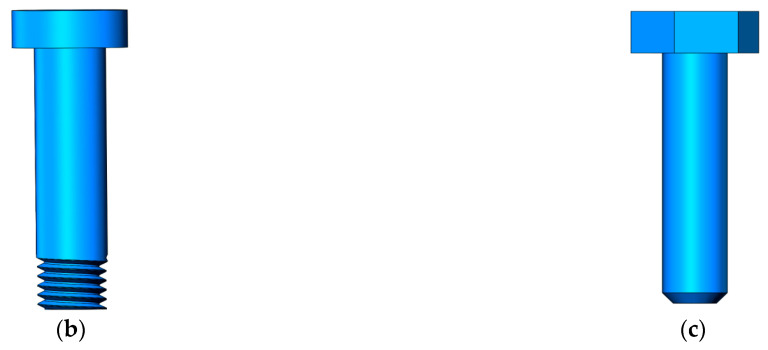
Structure of bolted joints under mixed loads. (**a**) Simplified bolt assembly, (**b**) with lifting angle bolts, (**c**) without lifting angle bolts.

Pre-loading relaxation is prone to lead to the functional failure of the bolt structure. It affects safety and reliability, and researchers have conducted many studies on the relaxation of pre-loading [9,10,11,12]. Yang [13] assumed that the spring stiffness is proportional to the magnitude of the force and constructed a model that can assess the pre-loading relaxation effect. Wang [10] used the cooling method to set the bolt pre-loading and investigated the effects of factors such as the friction coefficient and excitation replication on self-relaxation. Shen [14] established a wear model based on the secondary development of ABAQUS 2016 software, and the results showed that the pre-loading relaxation phenomenon can be predicted by fatigue damage theory and the wear model. At present, scholars have carried out more studies on the mechanisms, calculations, monitoring, and prevention of pre-loading relaxation [15,16,17,18,19], especially for analysis based on finite element software. However, there are fewer studies on the complex working conditions of high-precision instruments such as complex equipment. In the process of storage, transportation, and work of complex equipment, complex equipment may have impact, slip, and so on. These situations can generate large tangential displacements on the equipment, as shown in Figure 1. Therefore, it is necessary to predict the bolt pre-loading in this process. The existing methods for monitoring pre-loading mainly include the strain gauge sensor method [20,21], piezoelectric impedance method [22,23], ultrasonic method [24,25], and image recognition method [26,27,28]. These four methods are widely used in the fabrication of a wide range of physical field sensors. In particular, they play an important role in the fields of force measurement, weighing, and pressure sensors. However, such methods are complicated to implement and cannot monitor the change of pre-loading values in real time. Therefore, there is a need to build mathematical functions that can characterize the degree of pre-loading relaxation to solve the problem of unpredictable pre-loading decay during bolt operation.

In this paper, high-strength alloy nickel steel material is used as the basis from a safety point of view for large and complex equipment. The pre-loading experiment was designed and carried out. Nickel steel plates with a high strength, high hardness, and corrosion resistance are widely used in a variety of important complex equipment such as aircraft engines. However, due to its low filling factor, this material is less frequently used in existing common equipment, and there are fewer studies on nickel steel materials in the field at present. Therefore, this paper focuses on nickel steel structures in complex equipment, with a Z-shaped flat plate used to simplify the complexity of the equipment. The relationships between the mechanical properties of fasteners based on the ISO 898-2:2022 specifications are studied [29]. Conducting experiments at ambient temperatures from 10 °C to 35 °C ensures the physical properties of the screw and nut. The relationship between the pre-loading and maximum external tightening torque of M8 external hexagonal bolts was studied, and different pre-loading measurement methods and bolt tightening methods were analyzed. Pre-loading relaxation experiments were designed, and torque coefficient calibration between different bolts was carried out. The effects of different waveforms, surface roughnesses of nickel steel plates, tangential displacement frequencies, bolts, torques, and tangential cyclic displacements on pre-loading relaxation were studied. Parameter identification was carried out for different tangential displacements and initial tightening. An allometric model function and nine-stage polynomial function expression that could describe the degree of pre-loading relaxation were constructed. These can provide theoretical support for the safety and reliability of large and complex equipment.

## 2. Methods

### 2.1. Theoretical Formula for Calculating Pre-Loading

The total torque acting on the bolt structure is the sum of the friction torque produced by the threaded pair and the friction torque produced between the nut, which is as follows [30]:

(1) $$N=N_{1}+N_{2}$$

where $N_{1}$ and $N_{2}$ are calculated using the following formula:

(2) $$N_{1}=\frac{1}{2}\times F_{y}\times d_{2}\times \tan(\alpha +\beta )$$

(3) $$N_{2}=\mu_{w}\times F_{y}\times r_{d}$$

where $F_{y}$ is the bolt pre-loading, $d_{2}$ is the thread center diameter, α is the thread rise angle, β is the equivalent friction angle, $\mu_{w}$ is the friction coefficient of the inner surface of the nut, and $r_{d}$ is the equivalent friction radius.

The angle of thread rise is small in relation to the equivalent friction angle and can therefore be approximated as follows:

(4) tan(α+β)=tanα+tanβ

The equivalent friction radius can be expressed as follows:

(5) $$r_{d}=\frac{1}{3}\times \frac{\left({d_{1}}^{3}-{d_{0}}^{3}\right)}{{d_{1}}^{2}-{d_{0}}^{2}}$$

where $d_{1}$ is the maximum outer diameter of the nut and $d_{0}$ is the diameter of the bolt hole.

Therefore, the relationship between the tightening torque and pre-loading can be expressed as follows:

(6) $$N=\frac{1}{2}\times F_{y}\times d_{2}\times \tan(\alpha +\beta )+\frac{\mu_{w}\times F_{y}}{3}\times \frac{\left({d_{1}}^{3}-{d_{0}}^{3}\right)}{{d_{1}}^{2}-{d_{0}}^{2}}$$

The equation can be simplified as follows:

(7) $$N=K_{2}F_{y}d$$

where $K_{2}$ is the torque coefficient to be checked and d is the diameter of the bolt.

When the bolt is tightened, pre-loading should be controlled within a reasonable range. Too much pre-loading can lead to material fracture, failure, and yielding. Too little pre-loading can lead to reliability and safety problems in the equipment. The maximum tightening torque within the elastic range should be taken into account when assembling the bolt to the object to be connected to prevent equipment problems caused by excessive torque. Pre-tightening stress includes tensile stress σ and shear stress τ, which are calculated as follows:

(8) $$\sigma =\frac{F_{y}}{A_{s}}=\frac{4F_{y}}{\pi {d_{c}}^{2}}$$

(9) $$\tau =\frac{16N_{1}}{\pi {d_{c}}^{3}}$$

where $A_{s}=\frac{\pi }{4}{\left(d-0.9382P\right)}^{2}$ is the stress cross-section area, *P* is the pitch, and $d_{c}=\sqrt{\frac{4A_{s}}{\pi }}$ is the equivalent diameter.

Therefore, the relationship between the tangential and tensile stresses in the bolt can be expressed as follows:

(10) $$\frac{\tau }{\sigma }=\frac{2d_{2}\tan(\alpha +\beta )}{d_{c}}$$

According to the empirical formula, when the diameter of the bolt is less than 64 mm, it can be approximated as $d_{2}\approx 1.12d_{c}$. When the angle of thread rise and the equivalent friction angle is small, it can be approximated as tan(α+β) = 0.19. Therefore, it can be concluded that $\frac{\tau }{\sigma }\approx 0.43$.

The following is known from the theory of the fourth strength:

(11) $$\sigma_{\max}=\sqrt{\sigma^{2}+{\left(3\tau \right)}^{2}}$$

This can be solved as $\sigma_{\max}\leq 0.79\sigma_{s}$, where $\sigma_{s}$ is the yield strength. In the elastic range, the theoretical maximum bolt pre-loading can be expressed as follows:

(12) $$F_{y\max}=\sigma_{\max}A_{s}=0.79\sigma_{s}\times \frac{\pi }{4}{d_{c}}^{2}=0.1975\sigma_{s}\pi {d_{c}}^{2}$$

The theoretical maximum tightening torque can be found by bringing the formula into Equation (6) as follows:

(13) $$N_{\max}\approx 0.1975\times \pi {d_{c}}^{2}\sigma_{s}\left[\frac{d_{2}\left(\tan(\alpha +\beta )\right)}{2}+\mu_{w}r_{d}\right]$$

A GB/T 5783-2016 standard [31] 8.8 grade M8 external hexagonal bolt is used to calculate the maximum pre-loading and maximum torsion gauge. In this paper, the friction coefficient of the nickel steel flat plate is taken as 0.2, and its basic parameters are shown in Table 1. According to Equations (12) and (13), its theoretical maximum pre-loading and maximum torque can be calculated as 18,881 N, 34.3 Nm. It conforms to the design specifications and provides theoretical support for the selection of initial torque in the following.

### 2.2. Experimental Design of Pre-Loading Relaxation

#### 2.2.1. Main Experimental Equipment

**Figure 2:**
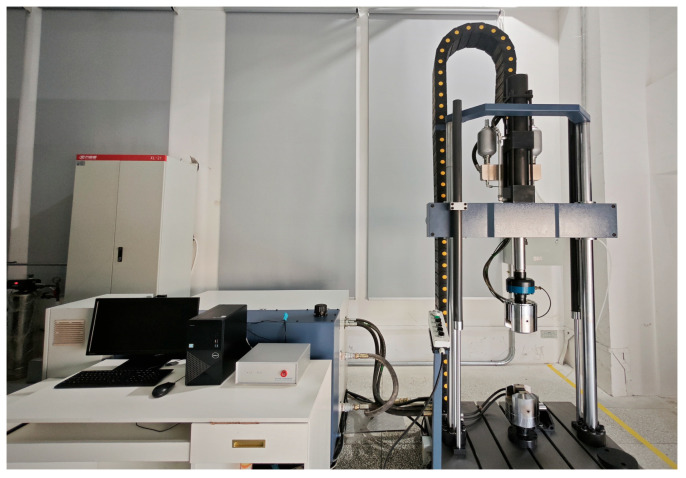
Starring experimental machine.

We used the 50 kN electro-hydraulic servo fatigue testing machine produced by Xi’an Tongsheng Instrument Manufacturing Co. (Xi’an, China). The machine can be applied to all kinds of components, materials, and dynamic and static mechanical properties tests. The main technical indicators are shown in Table 2. We used EVOTest 2.1.1.0 software to control the various performance parameters of the universal testing machine. The software is an external control system that can be close-loop controlled. Its test machine contains a variety of control methods, which can provide different kinds of stresses, strains, speeds, displacements, forces, and other external load application methods. The machine uses a fuzzy proportion integration differentiation (PID) control algorithm to regulate the loading process, which is able to obtain a high control accuracy. The universal experimental system is shown in Figure 2.

Due to the long-term operation of the experimental machine, the hydraulic oil is at a high temperature for a long time, and the rated temperature of the universal experimental machine is 55 °C, which needs to be water-cooled. This experiment uses an in-line water cooler, which is rated at 22 °C, and it uses softened water to circulate the hydraulic oil for cooling. The oil pump of the universal experimental machine is started using the EVOTest software. The locking cylinder is loosened, and the upper beam is adjusted to a suitable position. The cylinder is locked, the nickel steel flat plate is clamped in the upper and lower fixtures, and the experimental parameters are adjusted using the software.

#### 2.2.2. Main Measuring Devices

(1) Ring force sensor

In this paper, the strain gauge sensor method is selected to implement the monitoring of pre-loading recession values. Bengbu Jinnuo Sensor Co., Ltd. (Bengbu, China) Produced the JHBM-4 ring force sensor, and its specific parameters shown in Table 3.

**Figure 3:**
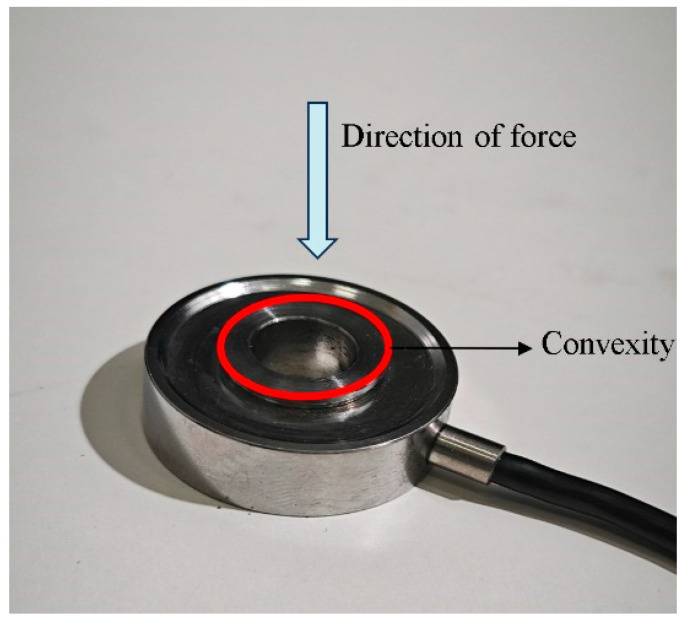
Working principle of the ring force sensor.

The ring force sensor is subject to bolt-induced pressure on the hub tab, as shown in Figure 3. In the experiment, it was placed in the middle surface of the bolt and the nickel steel plate to ensure that the convex surface was facing the bolt to enable it to accurately detect the bolt pre-loading.

(2) Intelligent display instrument

**Figure 4:**
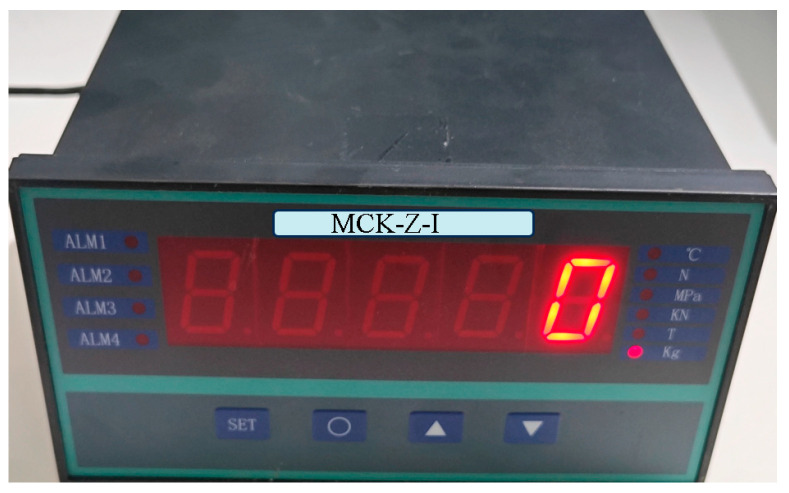
MCK-Z-I intelligent meter.

The ring force measuring sensor was connected to MCK-Z-I intelligent instrument as shown in Figure 4. It received signals from the sensor and generated electrical signals, and its specific parameters are shown in Table 4.

Before the experiment, the instrument needs to be calibrated for zero and display. Zero calibration requires entering the calibration mode and setting the value without preloading as the zero point, while display calibration needs to be based on the following calibration formula:

(14) $$k_{1}=\frac{X_{1}}{X_{2}}\times k_{2}$$

where $k_{1}$ is the calibration coefficient to be set, which ranges from 0.0010 to 9.9999; $k_{2}$ is the initial calibration coefficient rated at 1; $X_{1}$ is the value to be displayed; and $X_{2}$ is the currently displayed value.

Five-kilogram and ten-kilogram weights were placed on top of the ring force transducer tabs. The steady-state value was recorded in the intelligent display meter. The calibration coefficients were calculated to be set by the formula, and the coefficients were re-entered into the meter to complete the calibration. The calibrated meter was connected to a computer via the conversion cable, and the digital transmitter communication software was used to monitor and record the pre-loading recession value in real time.

(3) High-precision digital torque spanner

**Figure 5:**
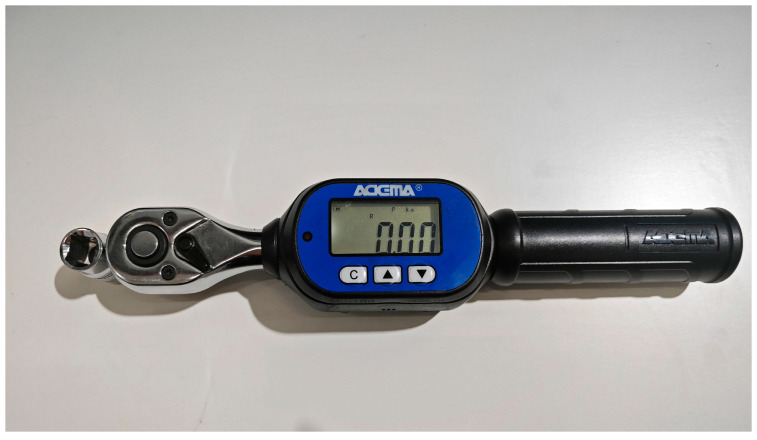
Torque spanner.

The tightening process of the bolt structure is divided into three stages. First, the bolt is not in contact with the plate, and its pre-tightening force is close to 0. Secondly, when the bolt head and nut are close to the plate, the pre-loading increases continuously. Finally, when the bolt reaches the yield limit and continues to tighten, pre-loading and torque will decrease, which may lead to the bolt fracturing. In large, complex equipment, bolt tightening methods are usually the torque method [32,33] and torque-angle method [34]. In this paper, the torque method is used to apply torque to obtain pre-loading, and the high-precision digital display torque wrench produced by Idema Company is selected, as shown in Figure 5. When tightening, the plate should be fixed, and the bolt head should be fixed at the same time to prevent experimental errors caused by the two. The torque wrench used in this experiment has a measuring range of 0~60 Nm, and its working life can reach 10,000 times. The accuracy can reach ±2% when tightening a bolt and ±2.5% when unloading a bolt. The target torque value can be preset, and the alarm for reaching the preset torque value improves the precision of the applied torque.

#### 2.2.3. Preparation of Laboratory Supplies

**Figure 6:**
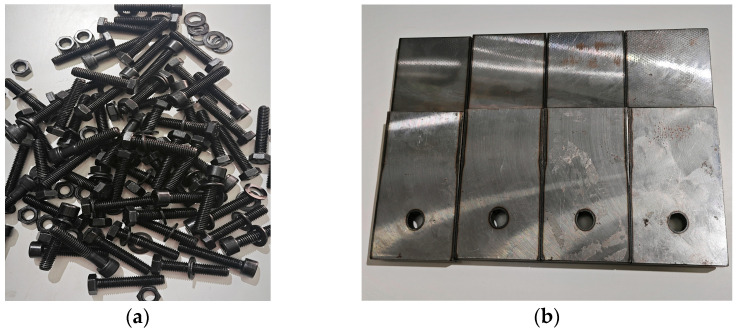
Experimental consumables. (**a**) Bolts, (**b**) nickel steel plate.

We selected national standard 8.8-grade M8 external hexagonal bolts and internal hexagonal bolts and 12.9-grade M8 external hexagonal bolts and internal hexagonal bolts as the specimens. Bolts with obvious defects were excluded before the test. To prevent damage to the threads, each bolt was loaded and unloaded only once during the experiment. Complex equipment in the complex structure is not convenient for the study of the pre-loading. Therefore, this paper simplifies the nickel steel high-strength material into a Z-shaped plate. This structure allows the bolt to be stressed at the center during pre-loading work, eliminating the effects of torque on the bolt, as shown in Figure 6. The simplified nickel steel flat plate can accurately simulate the force on complex equipment when the bolt is acting [35].

**Figure 7:**
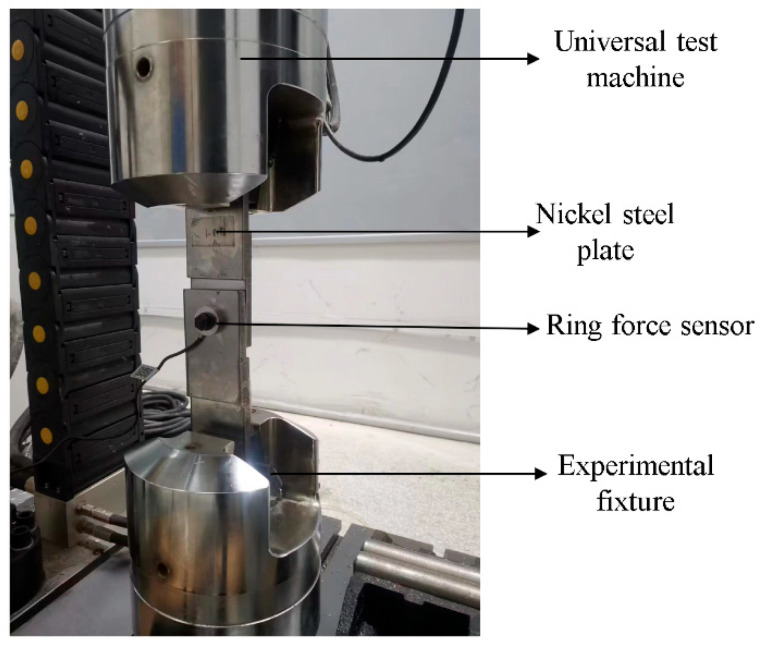
Overall assembly of the experiment.

The nickel steel flat plate was fixed in the fixture in the universal testing machine, and the overall assembly is shown in Figure 7. The ring force transducer was placed between the screw and the nickel steel flat plate, and the predetermined torque was applied using a torque spanner. Before the experiment, the tangential force and displacement in the universal testing machine were adjusted to zero. The ring force sensor was placed between the nut and the nickel steel flat plate. The pre-loading change value was read through the intelligent display meter connected to the sensor. The tangential load was applied using the EVOTest software on the computer side. The tangential cyclic load can be applied with a sine wave and half-sine wave. The computer and the intelligent display meter can record the real-time change value.

Before the pre-loading relaxation experiment started, the torque spanner and intelligent display instrument needed to be calibrated to verify the torque coefficient of the bolt to ensure the accuracy and feasibility of the experimental instrument. In this paper, the torque coefficient was calibrated for 8.8-grade M8 external hexagonal bolts and internal hexagonal bolts and 12.9-grade M8 external hexagonal bolts and internal hexagonal bolts. The maximum torque was calculated to be 34.3 Nm for 8.8-grade M8 bolts and 44 Nm for 12.9-grade bolts. A smaller torque leads to the bolts not reaching the pre-tensioning effect, and a larger torque leads to the bolts failing, which affects the subsequent experimental tests. Therefore, this paper selected 4–28 Nm torque. In the form of incremental selection of five torque values, can make the torque cover the range of the standard.

**Figure 8:**
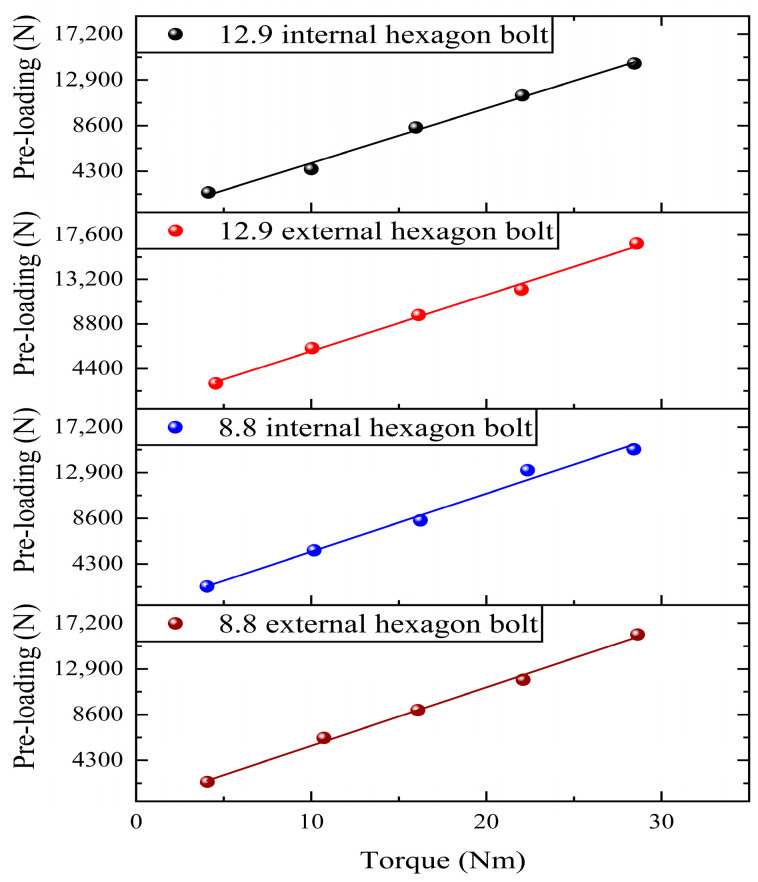
Torque coefficient calibration.

The nickel steel flat plate was fixed in the universal experimental machine, and a spanner was used to fix the nut to prevent the nut from loosening during tightening. The required torque was set using a torque spanner, and the pre-loading was applied in the form of a tightening screw. Five torques of 4 Nm, 10 Nm, 16 Nm, 22 Nm, and 28 Nm were applied to each set of bolts in turn. Each bolt was applied only once for loading and unloading to avoid the problem of reduced bolt pre-loading due to repeated tightening. Each set of experiments was repeated three times, and the average value was taken to circumvent the effects of experimental chance. The experimental results are shown in Figure 8, and the linear expression of the torque coefficients is shown in Table 5. Since the slope of the fitted curve is large, fluctuations at smaller slopes can affect the intercept to produce larger fluctuations. The value of Pearson’ s r ranges from −1 to 1. When the value is greater than 0.8, it can indicate that the original data are strongly correlated with the fitted curve. The closer that the R-squared and adjusted R-squared values are to 1, the better the fit. The 8.8 external hexagon bolts fit best, and 8.8 internal hexagon bolts fit worse. However, all of them reached more than 98.5%, indicating that the overall fitting effect of the curves was good. Its slope was stable within a certain range.

The torque coefficient is affected by the tightening speed of the nut, the presence or absence of shims, the thickness of shims, the presence or absence of lubrication, the material, and the ambient temperature. Existing studies have proved [36] that the torque coefficients of the above four types of bolts range from 0.2 to 0.45 with the installation of shims based on the calculation of the torque using Equation (7). Under the roughness Ra1.6 of nickel steel plate specimens, the torque coefficient of 8.8-grade M8 external hexagonal bolts ranges from 0.2360 to 0.2525, and the torque coefficient of 8.8-grade M8 internal hexagonal bolts ranges from 0.2312 to 0.2615. The torque coefficient of 12.9-grade M8 external hexagonal bolts ranges from 0.2374 to 0.2576, and the torque coefficient of 12.9-grade M8 internal hexagonal bolts ranges from 0.2504 to 0.2722. The torque coefficient of 8.8-grade M8 external hexagonal bolts is small, and the torque coefficient of 12.9-grade M8 internal hexagonal bolts is the largest. Their torque coefficients are all within a reasonable range, proving the accuracy of the experimental equipment and measurement methods in this paper. 

### 2.3. Bolt Performance Prediction Methods

The bolts were subjected to mixed loads during operation, which led to a relaxation of the pre-loading. However, it was not possible to monitor the pre-loading again using instruments. Therefore, this paper proposes to characterize the pre-loading relaxation law using a mathematical function. The variation of pre-loading under different tangential cyclic loads is predicted. Among the fitting methods are the allometric model function [37], high-order polynomial function [38], and Gaussian function [39].

(1) Allometric model function

The allometric model function is a method for obtaining optimal parameter estimates in the form of power functions. Built on the basis of a “hierarchy of universes and developments”, it allows for quantitative access to models and behaviours. The function is used to describe a curve that grows or decays in the form of a power exponent. The basic formula is as follows:

(15) $$y=a\cdot x^{b}$$

where x and y are the independent and dependent variables, respectively, and a and b are the coefficients given by the model.

When x is small, the dependent variable changes more significantly, and its slope changes more. When x is large, the dependent variable and slope are regionally stable. It is in line with the law of pre-loading relaxation, so it can be used to predict the performance assessment of pre-loading relaxation.

(2) Higher-order polynomial function

The higher-order polynomial fitting algorithm is a method for approximating data points using a polynomial function that minimizes the error between the fitted function and the actual data points. The basic idea is to improve the accuracy of the fit to the data by increasing the order of the polynomial. The general form of high-order polynomial fitting is as follows:

(16) $$y=a_{0}+a_{1}x+a_{2}x^{2}+\ldots +a_{n}x^{n}$$

where $a_{0}$, $a_{1}$, $a_{2}$, …, $a_{n}$ denote the polynomial coefficients and n denotes the order of the polynomial. The optimal values of the polynomial coefficients can be solved using mathematical statistical methods such as least squares to obtain an optimal fitting function.

It is important to note that high-order polynomial fitting is prone to overfitting problems. The fitting function is too complex and too sensitive to the data, resulting in a poor fit. To avoid the overfitting problem, regularization methods can be used to optimize the high-order polynomial fitting. In practice, it is important to choose the appropriate polynomial order according to the complexity of the data. Low-order polynomials can be chosen for simple data, while high-order polynomials are required for complex data. At the same time, the fitting results must be evaluated and tested to ensure the validity and reliability of the fitted function. Higher-order polynomials can be fitted directly without a specific physical model. As the number of times increases, it will show the phenomenon that the degree of fit also becomes higher. However, when the number of times is high to a certain degree and then continues to increase, the number of times will exhibit the overfitting phenomenon. After pre-calculation and deduction, the polynomial is the highest degree of fit when the polynomial is 9 times.

(17) $$y=a_{0}+a_{1}x+a_{2}x^{2}+a_{3}x^{3}+a_{4}x^{4}+a_{5}x^{5}+a_{6}x^{6}+a_{7}x^{7}+a_{8}x^{8}+a_{9}x^{9}$$

The specific steps of the fitting process are as follows: use the readtable function to read the data in the file and set the fitting order n from 1 to 9 in a loop. Use the polyfit function to fit the read data, then solve the parameters to judge the degree of fit.

(3) Gaussian function

Gaussian functions are widely used in statistics to express the normal distribution. The function approximates the set of data points for prediction. The function is characterized as a bell-shaped curve with a multinomial Gaussian function using the following formula:

(18) $$y=a_{1}e^{-{\left(\frac{x-b_{1}}{c_{1}}\right)}^{2}}+a_{2}e^{-{\left(\frac{x-b_{2}}{c_{2}}\right)}^{2}}+a_{3}e^{-{\left(\frac{x-b_{3}}{c_{3}}\right)}^{2}}+a_{4}e^{-{\left(\frac{x-b_{4}}{c_{4}}\right)}^{2}}+a_{5}e^{-{\left(\frac{x-b_{5}}{c_{5}}\right)}^{2}}$$

where a is the height of the peak of the curve, b is the center of the peak region, and c characterizes the width of the bell curve.

The Gaussian function makes predictions based on historical statistics. This creates a predictive model that describes subsequent developments. The above formula is only a common Gaussian function curve fitting form. The specific application may be based on the needs of the problem and the characteristics of the data to choose the appropriate form of the function. In addition, in order to obtain the best fitting effect, it is usually necessary to optimize the parameters using the least squares method after a large number of analyses of the pre-loading data.

In order to verify the prediction effect of the three functions, this paper carried out relaxation experiments on 8.8-grade M8 external hexagonal bolts and internal hexagonal bolts, as well as 12.9-grade M8 external hexagonal bolts and internal hexagonal bolts. The three functions were fitted to the obtained data, and the fitting parameters are shown in Table 6.

The accuracy of most of the three fitting functions was above 0.98, which indicated a high fitting effect. However, the Gaussian function had 15 unknown parameters and the most complicated structure. Therefore, for the sake of the simplicity of the subsequent calculations, the Gaussian function is discarded in this paper. The allometric model function, a the nine-stage polynomial function, is used to predict the performance of the bolt.

## 3. Results and Discussion

### 3.1. Multiple Factor Impact Analysis

#### 3.1.1. Different Surface Roughness Analysis

**Figure 9:**
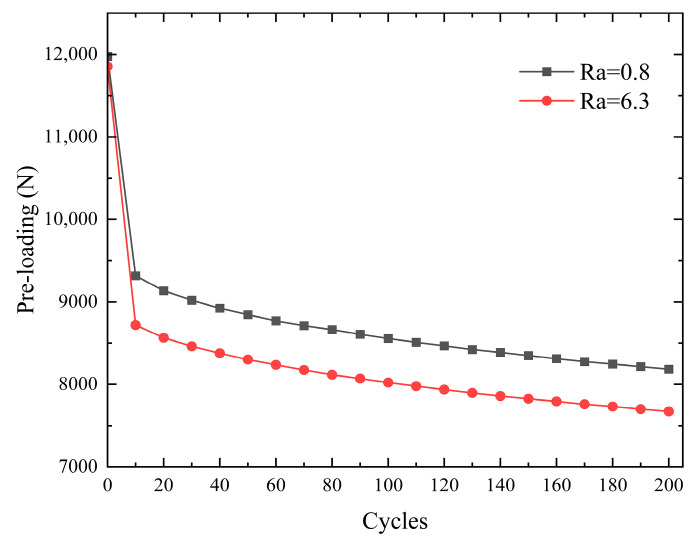
Pre-loading relaxation at different surface roughnesses.

Different surface roughnesses of nickel steel flat plates affect the pre-loading of the bolt connection structure. To further analyze the effects of roughness on the pre-loading relaxation of bolted joint structures, the controlled variable method was used to study 8.8-grade hexagonal bolts acting in nickel steel flat plates with Ra0.8 and Ra6.3 roughnesses. Experimentally, a tightening torque of 22 Nm was applied, and a tangential cyclic displacement control of 0.5 mm with a half sine wave was used. Two hundred groups of repetitive experiments were carried out, and the value of pre-tightening force in the MCK-Z-I intelligent meter was recorded every 10 groups. The experimental results are shown in Figure 9. The greater the roughness of the nickel steel plate, the more the pre-loading decayed. Its decay rate was approximately the same, which was the same as the results of the literature [40], proving the correctness of the experimental design of this paper.

#### 3.1.2. Multiple Cycle Frequency Loading

**Figure 10:**
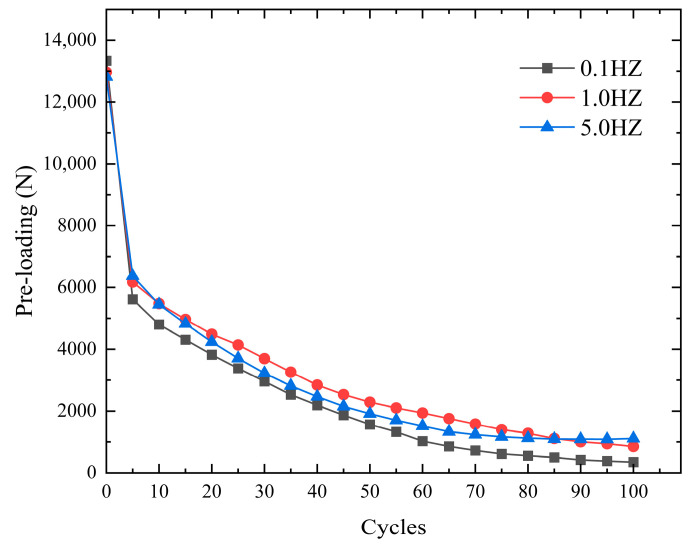
Comparison of three cycle frequencies.

In order to investigate the pre-loading loosening behavior of the bolted joints with half sine wave displacement control at different loading frequencies, three (0.1 Hz, 1.0 Hz, 5.0 Hz) cyclic frequency experimental conditions were selected. A nickel steel plate with Ra1.6 roughness was used to control the tangential cyclic displacement of 1 mm, and 100 sets of loosening experiments were carried out. The value of pre-loading in the oscilloscope was recorded every 5 groups of cycles, and the experimental results are shown in Figure 10. The most loosening of the bolt pre-loading occurred under lower-frequency cyclic tangential loading. With higher-frequency cyclic tangential loads, the bolt pre-loading loosening was less. The contact time between the threaded sub and the nickel steel flat plate screw hole was longer under low-frequency loading. The connection interface was stressed for a longer period of time, resulting in more loosening of the bolt pre-loading.

**Figure 11:**
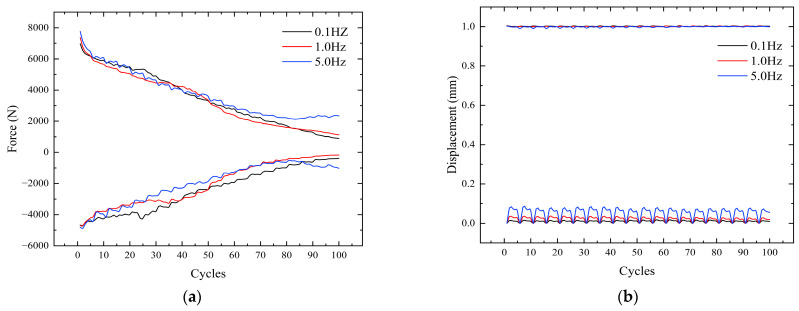
Variation curves at different frequencies. (**a**) Force change curve. (**b**) Displacement change curve.

As the number of cycles increases, the bolt gradually begins to loosen, as shown in Figure 11a. The force required to reach a tangential displacement of 1 mm gradually decreases. This leads to the gradual loosening of the bolt, which affects the safety of the equipment. The upper peak of displacement can reach 1 mm stably, but there is some fluctuation in the lower peak. The existence of a gap between the nickel steel plate and the bolt leads to instability in the displacement when the plate returns to the initial position, as shown in Figure 11b. This fluctuation is unavoidable because the gap between the two cannot be eliminated.

#### 3.1.3. Differential Study of Waveform Control

**Figure 12:**
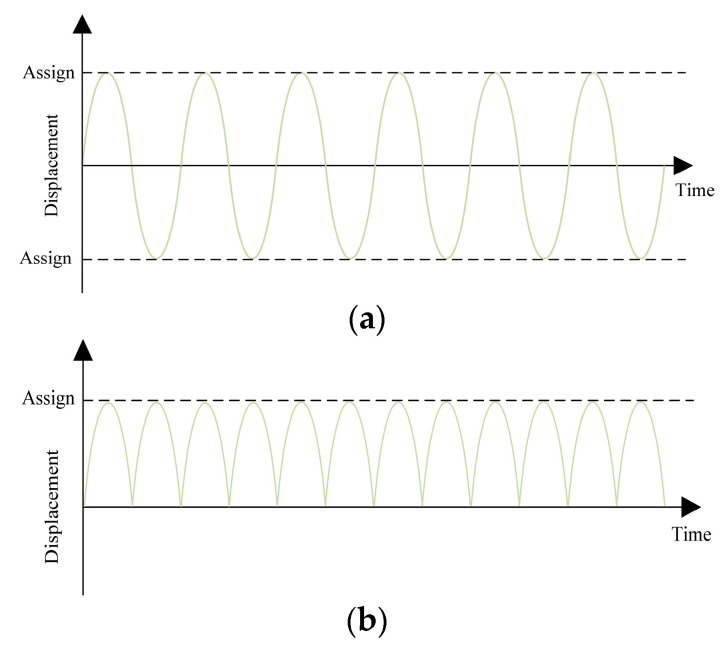
Two types of waveforms. (**a**) Sine wave, (**b**) half sine wave.

The universal experimental machine can be equipped with sine and half-sine waves with different tangential cyclic displacements, as shown in Figure 12. Under waveform control, the rising phase of the curve is the universal testing machine stretching phase. When the displacement value reaches the set value, the curve begins to fall, which is the universal laboratory machine compression stage.

**Figure 13:**
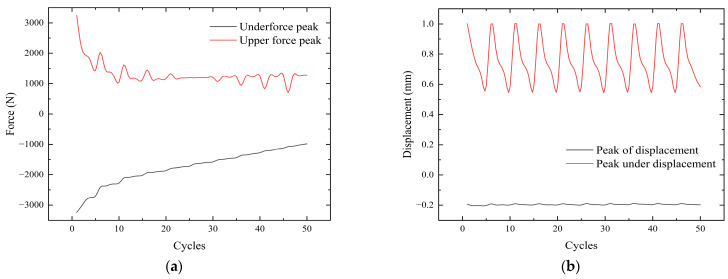
Waveform curve under sinusoidal waveform control. (**a**) Force change curve. (**b**) Displacement change curve.

Based on 8.8-grade M8 hexagonal bolts carried out under sinusoidal wave control in the form of tangential cyclic displacement, when the machine reaches a set displacement, a certain tensile force is generated. However, this force presents a non-linear variation and cannot ensure a single variable for the experiment. Therefore, the use of a displacement signal ensures that the flat plate pulls the same displacement. In this experiment, the center position is 0 mm, the amplitude is 1 mm, the vibration frequency is 1 Hz, the target number of cycles is 500, and the force/displacement cycle is shown in Figure 13. In the initial vibration stage, the lower peak value of displacement was stable at about 0.2 mm, and the upper peak value of displacement showed fluctuations between 0.584 and 1 mm. Neither reached the set value, which led to changes in the tension of the universal testing machine. The reason for this is that the hole diameter of the nickel steel plate is 10 mm, the bolt diameter is 8 mm, and there is an unavoidable gap between the two. The buckling phenomenon occurs when reciprocating displacement is applied to the universal testing machine. The change in displacement will have repeated impacts on the center gap, which in turn causes the inaccuracy of the results. Therefore, the displacement signal of the sine wave control did not meet the set standard.

**Figure 14:**
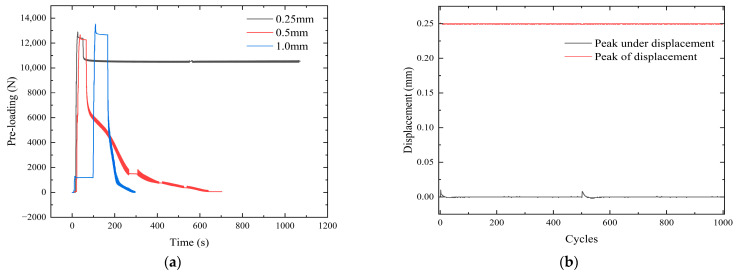
Waveform curve under half sine wave control. (**a**) Half sine wave displacement control. (**b**) Displacement change rule.

Adjusting the waveform to a half-sine eliminates the effects of simultaneous tensile and compressive loading of the nickel steel plate. Its displacement stability is shown in Figure 14. The torque of 22 Nm was applied in the three groups of experiments, and the initial pre-loading values were 12,398 N, 12,285 N, and 12,696 N, respectively. Due to the error of the experiment, the pre-loading fluctuates in a certain range. The total number of cycles at a tangential 1.0 mm displacement was 130. However, at 73 cycles, the bolt had loosened and had no tightening effect. The total number of cycles for a tangential displacement of 0.5 mm is 500, whereas for 400 cycles the pre-loading is less than 100. At 400 cycles, the value of pre-loading is less than 100. A total of 1000 cycles is required for 0.25 mm tangential displacement, and the pre-loading remains in the stable range. At the end of the experiment, 10,539 N of pre-loading remained, with a loss of only 15%. The experimental results show that the bolts remained robust under a small displacement and loosened faster under a large displacement.

### 3.2. Study on Relaxation Law of Bolt Pre-Loading

#### 3.2.1. Analysis of Relaxation Efficiency of Different Bolts

**Figure 15:**
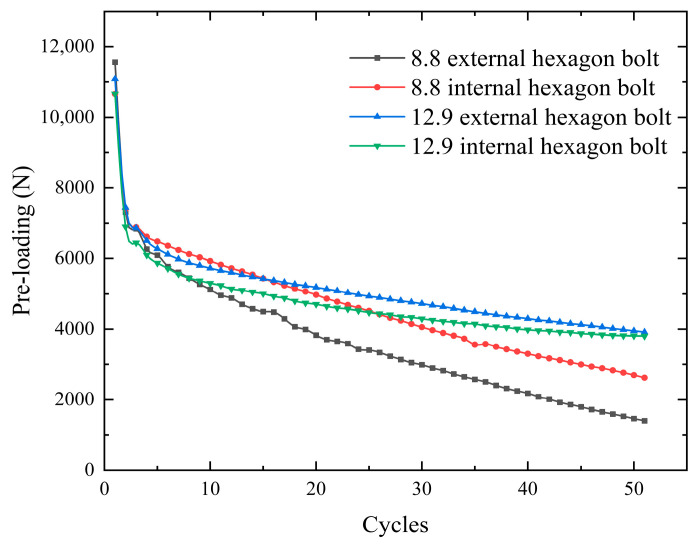
Pre-loading relaxation of different bolts.

In order to more finely study the changes in pre-loading relaxation under bolt vibration conditions, this paper carries out a manual control of a universal testing machine in order to stretch and compress a nickel steel flat plate. This operation eliminates the effects of the half-sine wave. Based on nickel steel flat plates with a Ra1.6 roughness, the effects of different bolt strengths and different bolt head contact areas on pre-loading relaxation are investigated. The median of this experiment is 0 mm, the amplitude is 1 mm, the vibration frequency is 1 Hz, and the target number of cycles is 50 times. The relaxation law of pre-loading is shown in Figure 15, and the specific relaxation parameters are shown in Table 7. High-strength bolts have high anti-loosening properties. Under the same bolt strength grade, the outer hexagon bolt loosens faster than the inner hexagon bolt. The contact area of the bolt head has a certain influence on the relaxation of pre-loading.

**Figure 16:**
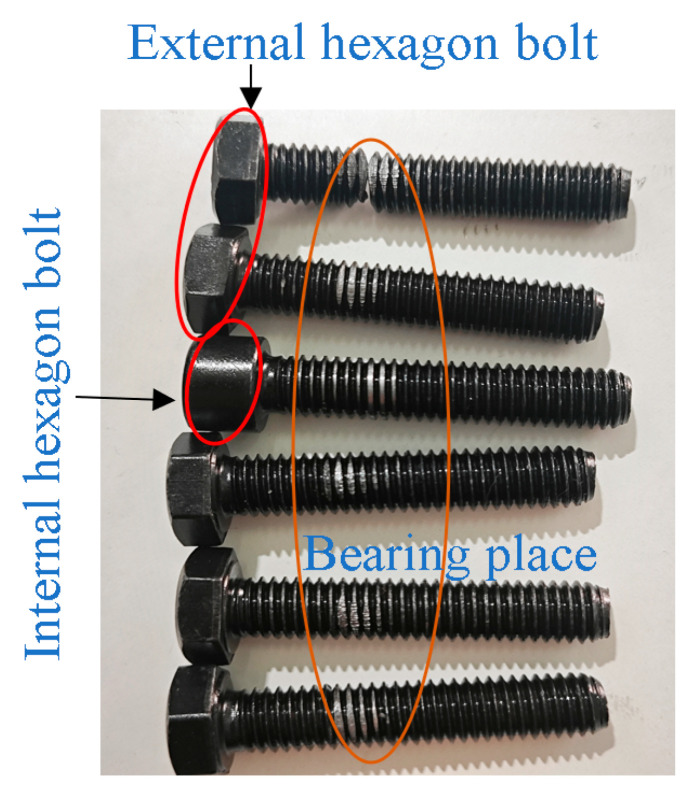
Degree of bolt damage.

The extent of damage to the threads of the different bolts is shown in Figure 16. The length of the bolt is 40 mm, and the severe wear is at the coupling of the upper and lower nickel steel plates. There is a gap at the coupling, and it is subjected to shear force, which results in a high degree of thread wear. The surface of the flat plate and the threads, the nut, and the screw are all in cross-scale phenomena. Under the control of a large displacement, after many cycles of vibration, it is easy to cause fractures here, which affect the safety performance of the equipment.

**Figure 17:**
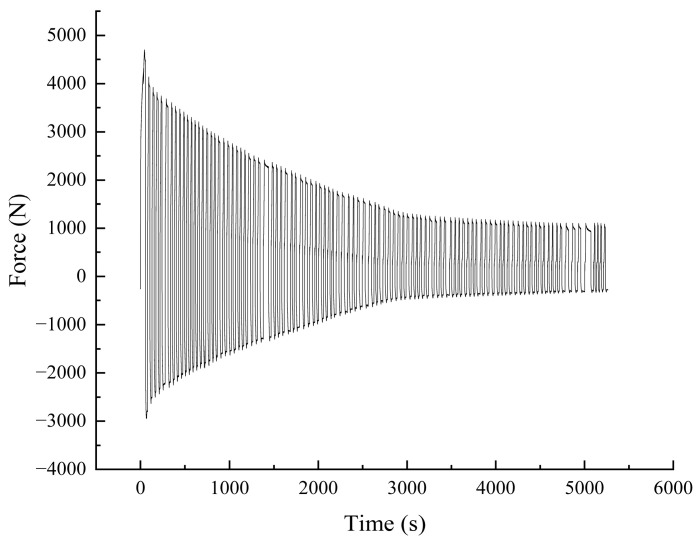
Force time variation relationship.

During the tangential load loading process, the tangential tension of the universal testing machine versus time is shown in Figure 17. As the number of loading times increases, the force required to reach the set 1 mm tangential displacement gradually decreases. In the last cycle, the positive tension reaches 1085 N, which is 76.8% lower than the initial tension value. The negative pressure reaches 276 N, which is 90.6% lower than the initial pressure value, and the pressure attenuation is more. At this time, the bolt loosening degree is large, affecting the safety and reliability of the equipment.

#### 3.2.2. Effects of Different Tangential Displacements

**Figure 18:**
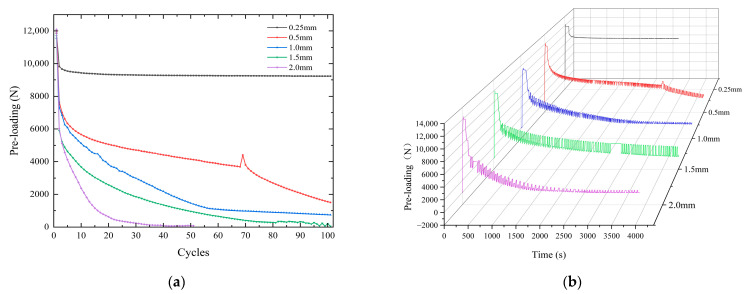
Pre-loading change rule under five kinds of tangential displacements. (**a**) Nodal variation curve of pre-loading cycle. (**b**) The whole process change curve.

A phase 22 Nm torque was applied to the same kinds of blots to study the bolt pre-loading relaxation law under 0.25 mm, 0.5 mm, 1.0 mm, 1.5 mm, and 2.0 mm tangential cyclic displacement. The tangential load vibration experiments were carried out 100 times to record the value of pre-loading when the tangential displacement was 0. The experimental results are shown in Figure 18a, and the whole process of bolt pre-loading changes is shown in Figure 18b. Under the action of a small load of 0.25 mm, the bolt can maintain a relatively good tightening effect. The pre-loading loss is only 22.0% after 100 cycles. With the increase in tangential displacement, the attenuation of bolt pre-loading increases. Under the action of a 2.0 mm tangential displacement, the bolt reached complete relaxation in only 50 cycles. In the first vibration cycle, the pre-loading was reduced by 50.2%, as shown in Table 8. It can be seen that under the action of a large tangential vibration load, the bolt pre-loading relaxation was faster. This affects the safety and reliability of equipment. In engineering practice, multiple bolts can be tightened in a nickel steel plate to avoid the impact of large load tangential displacement on complex equipment.

**Figure 19:**
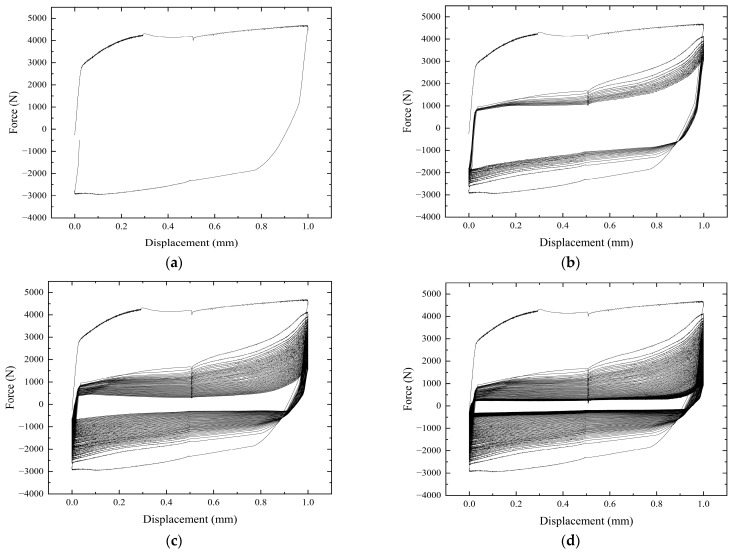
Hysteresis return line at a 1 mm displacement in tangential direction. (**a**) 1 cycle. (**b**) 15 cycles. (**c**) 50 cycles. (**d**) 100 cycles.

The hysteresis return line under a 1 mm tangential cyclic displacement is shown in Figure 19. Within a single cycle, the area enclosed by the curve is the energy dissipation value. As the cycle period increases, the curve gradually approaches the Y = 0 region, and its energy dissipation gradually decreases. The difference in energy dissipation between cycle 1 and cycle 2 is large, reflecting the large change in the value of bolt pre-loading in the next cycle. The maximum values of forces in different cycles in the figure are 4697 N, 3022 N, 1546 N, and 1030 N. The maximum force in 100 cycles is reduced by 78.1% compared to 1 cycle. The structure reaches the same tangential displacement, is subjected to a gradually decreasing maximum external tension, and has a lower degree of energy dissipation.

#### 3.2.3. Effects of Different Initial Tightening Moments

**Figure 20:**
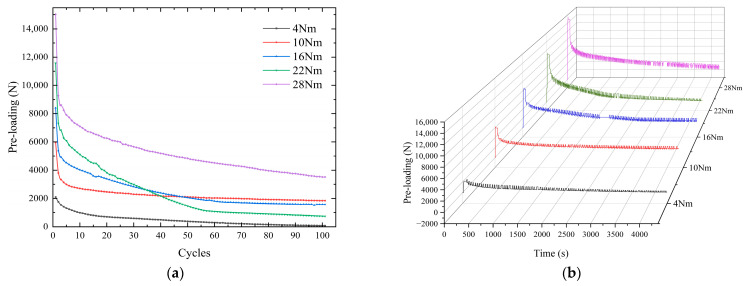
Pre-loading decay under different torques. (**a**) Nodal variation curve of pre-loading cycle. (**b**) Change curve of the whole process.

Based on a Ra1.6 roughness nickel steel plate, bolt pre-loading relaxation experiments was carried out under different torques (4 Nm, 10 Nm, 16 Nm, 22 Nm, 28 Nm). The magnitude of the pre-loading at the tangential cyclic 0 mm displacement was recorded. The experimental results are shown in Figure 20a, and the curve of the whole process of pre-loading relaxation is shown in Figure 20b. In the process of stretching and compression displacement, the pre-loading has a small fluctuation. The initial pre-loading is 2105 N, 5963 N, 8402 N, 11,567 N, and 15,027 N, respectively. After 100 cycles of tangential 1 mm displacement, the remaining pre-loading is 78 N, 1845 N, 1568 N, 742 N, and 3523 N, respectively, and the specific decay percentages are shown in Table 9. Under the large displacement of 1 mm, the pre-loading generated by the 4 Nm torque is small and does not reach the tightening effect, resulting in its faster loosening. The 10 Nm torque has a good tightening effect, and the loss of pre-loading is only 69.1% after 100 vibration cycles. However, between 10 and 22 Nm, after the first cycle, there is little difference in the decay efficiency of pre-loading among the three. After several cycles, with the increase in torque, the attenuation speed of pre-loading increases, and the bolts can maintain a good pre-loading performance under the action of 28 Nm.

**Figure 21:**
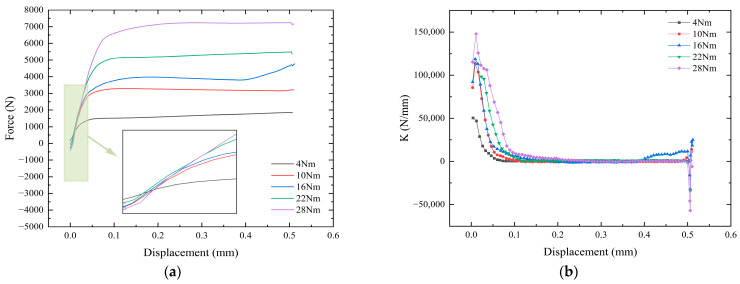
Energy change under different initial pre-loading conditions. (**a**) Backbone curve. (**b**) Stiffness degradation curve.

The backbone curve and stiffness degradation curve for a single stretch to 0.5 mm displacement are shown in Figure 21. The maximum force applied to the universal testing machine in the range of tangential displacement of 0–0.5 mm are 1852 N, 3285 N, 4770 N, 5486 N, and 7246 N. The increase in the initial torque of the bolts leads to an increase in the tensile force of the universal testing machine, but there is no linear multiplication.

The initial stiffness increases along with the increase in the tightening torque, respectively: 5.0438 × 10^4^ N/mm, 8.5500 × 10^4^ N/mm, 9.2083 × 10^4^ N/mm, 9.8404 × 10^4^ N/mm, 11.5200 × 10^4^ N/mm. The bolt stiffness will show a brief upward trend in the initial stage. As the tangential displacement increases, the stiffness gradually degrades. At the 0.5 mm tangential cyclic displacement, the bolt is subjected to shear action, and the stiffness begins to change.

The bolt pre-loading degradation law presents two stages. The first stage is a rapid decline in bolt pre-loading, and the second stage is a slow decline. The first stage is caused by excessive local stress caused by the steel plate extrusion bolt and excessive pressure on the annular support surface of the fastener head. At the same time, the material yields and undergoes plastic deformation, which in turn leads to the relaxation of bolt pre-loading. It can be easily obtained from the data analysis that the phase transition process occurs at about 5–10 cycles. The second stage is when the bolts are subjected to large loads in the tangential cycle, and the bolted structure is most prone to rotational loosening. The next most prone to loosening is torque loading, and axial loading is the least prone to loosening. It is worth noting that the occurrence of relative rotation is not instantaneous and requires some accumulation. Local sliding is caused by a long accumulation, so the second stage is a slow descent process.

### 3.3. Predictive Study of Bolt Pre-Loading Performance

#### 3.3.1. Performance Prediction under Multiple Displacements

**Figure 22:**
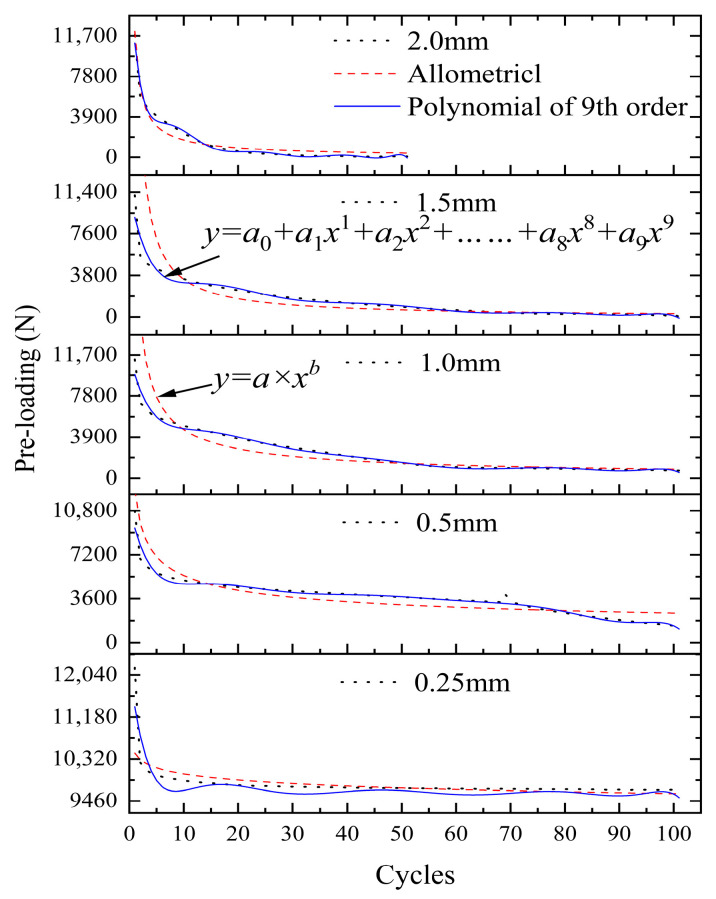
Mathematical function fitting for different tangential cyclic displacements.

The mathematical function of different tangential cyclic displacements is investigated for the case of a 22 Nm external tightening torque. The pre-loading relaxation law function is constructed using the allometric model function and the nine-stage polynomial function. The mathematical functions for five different tangential cyclic loads are shown in Figure 22. 

The Allometricl model function is in good agreement with the experimental values after several cyclic loadings. The fitting accuracies are 0.71719, 0.72726, 0.88981, 0.85451, and 0.94252, respectively. There are large deviations in the data in 10–40 cycles, and the deviations are small in other cycles. With the increase in the tangential cyclic displacement, the parameter a in the power function increases gradually, and the parameter b decreases gradually. For a small displacement of 0.25 mm, the maximum prediction error is 1.2%. In the case of a 1.5 mm large displacement, the maximum prediction error is 38.2%. There are obvious differences in the curve equations, indicating that the relaxation change rules of the pre-loading are different. The nine-stage polynomial function improves the fitting accuracy by increasing the order of the polynomial. The fitting accuracy under a 0.25–2 mm displacement is 0.90364, 0.96814, 0.98343, 0.95656, 0.98215, respectively. Its fitting accuracy is higher than the alometric model function. The fitting accuracy of the two functions is poor when the displacement load is 0.25 mm, mainly because the relaxation of the pre-loading is relatively slow and the region is gradually stable.

#### 3.3.2. Performance Prediction under Multiple Initial Torques

**Figure 23:**
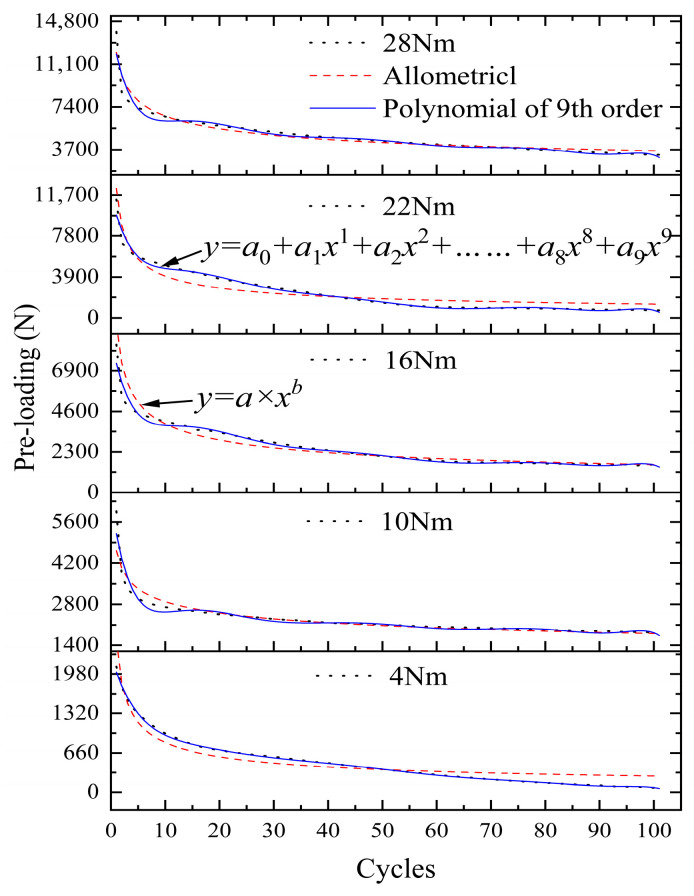
Mathematical function fitting for different initial pre-loadings.

Based on the 1 mm tangential cyclic displacement, the mathematical function expression of the change in different initial pre-loadings with the number of cycles is constructed. The pre-loading relaxation law function and the parameters in the discrimination function can be constructed using two methods. The results of parameter identification under different initial pre-loadings are shown in Figure 23. The fitting accuracy of the fitting function in 4–28 Nm are 0.88923, 0.89613, 0.90541, 0.87723, and 0.94667. There are deviations in 10–40 cycles. The 35-cycle deviation in the 4 Nm torque is 31.3%, but the difference in pre-loading is small, which is in the normal range. Parameter a shows fluctuating changes under different initial pre-loads, and parameter b is more stable. It shows that the initial torque only affects parameter a. Overall, the curve parameters are well identified. Under the same tangential cyclic displacement, the curve patterns are approximately the same. The fitting accuracies of the nine-stage polynomial function for 4–28 Nm are 0.99818, 0.93917, 0.97441, 0.98343, and 0.95481, respectively. The fitting effects are all above 95%, which have a good accuracy. However, its polynomial contains 10 parameters to be identified, while the allometric model function has only 2 parameters to be identified, which leads to more complicated results.

In Origin 2022, pre-loading data plots are constructed and fitted, and the parameters are identified by setting the function. Based on the least squares method, numerical simulation techniques were used to determine the appropriate parameters of the function. The objective function describes the variation of the data through the optimal parameters. In this case, the nine-stage polynomial is used to select the optimal value by increasing the order from 1–9. The fitting effect is judged by the goodness of fit and root mean square error. Combined with the above studies, the allometric model function fits the worst, at 71.7% for the small displacement condition. Other than that, the allometric model function and the nine-stage polynomial function can reach more than 85.5% and 90.4%. This proves the accuracy of this paper for the prediction of bolt pre-loading relaxation. 

Combined with the above studies, the bolt pre-loading relaxation under the action of tangential cyclic displacement can be divided into two stages. The first stage is the stage of rapid decrease of bolt pre-loading, and the second stage is the slow decrease process. The relaxation law in a specific case can be expressed by the allometric model function and nine-stage polynomial function. It can provide theoretical support for the safety and reliability of large and complex equipment.

## 4. Conclusions

Based on the problem that there are more bolt connection structures in complex equipment and the structure is prone to failure, this paper is based on the ring force transducer, universal experimental machine, and other equipment, with a simplified high-strength Z-shaped nickel steel plate used as the object of study. The relaxation law of bolt pre-loading for complex equipment is studied. The maximum tightening torque of the bolt was calculated, and the bolt pre-loading relaxation experiment was designed. The range of torque coefficients between bolt pre-loading and torque were calibrated, and they were all between 0.2312 to 0.2722, which was in line with the design range. The effects of different waveforms, nickel steel plate surface roughnesses, tangential displacement frequencies, bolts, torque magnitudes, and cyclic tangential displacements on pre-loading relaxation were investigated. A study of bolt performance prediction methods was carried out. The experimental results showed the following:

(1) The half sine wave has better stability. The rougher the nickel steel plate, the more pre-loading decay, but the decay rate is about the same. However, the rate of decay is approximately the same. The nickel steel plate of Ra6.3 is rougher than the plate of Ra0.8, with more more pre-loading attenuation, and the rate of decay is approximately the same. The contact time between the threaded sub and the screw hole of the nickel steel flat plate is longer under low-frequency loading. The connection interface is stressed for a longer time, resulting in more loosening of the bolt pre-loading. For the same bolt strength class, the outer hexagon bolts loosen faster than the inner hexagon bolts, and the high-strength bolts have a better stability.
(2) The pre-loading decay rate of the bolts increases as the tangential cyclic displacement increases. At a larger tangential cyclic displacement of 2 mm, the bolt is completely loosened and not tightened after 50 cycles of tangential vibration. At the smaller tangential cyclic displacement of 0.25 mm, the bolt loosened more slowly. After many cycles, it still maintained a good tightening effect. At a smaller initial torque, the bolt was not tightened. A 28 Nm torque had a better tightening effect, and between 10 and 22 Nm, after the first cycle, the three pre-loading decline efficiency was not significantly different. After several cycles, along with the increase in torque, the pre-loading decay rate increased.
(3) The bolt pre-loading degradation law shows two stages. The first stage is a rapid decline in bolt pre-loading, and the second stage is a slow decline process. The first stage is due to the steel plate extrusion bolt resulting in excessive local stress, and fastener head ring support surface pressure caused by excessive yielding of the material makes the material undergo plastic deformation. This in turn leads to relaxation of the bolted joint pre-loading. The second stage is due to the slow decline phase resulting in micromotor wear. Wear cannot be instantaneous and needs to accumulate gradually.
(4) Parameter identification of pre-loading relaxation curves was carried out. Mathematical functions that can express different tangential cyclic displacements and different initial pre-loadings were constructed. Under the small displacement condition, the allometric model function fit the worst, reaching 71.7%. Otherwise, the allometric model function was able to predict more than 85.5%, which required the use of least squares to identify the two unknown parameters. The nine-stage polynomial function fitting accuracy can reach more than 90.4%, which requires the use of least squares to identify 10 parameters. The complexity of the two is different, but both can better characterize the pre-loading relaxation law under specific conditions. The accuracy of this paper’s prediction of bolt pre-loading relaxation is demonstrated.
(5) In this paper, the pre-loading relaxation experiments of many kinds of bolts are carried out, and the study of relaxation based on the allometric model function and nine-stage polynomial function is carried out. In this paper, the bolt performance under the condition of a large tangential external load can be predicted. It is helpful to improve the safety and reliability of complex equipment, the aerospace industry, and other equipment, and to provide theoretical support for it.

## Figures and Tables

**Table 1:** Bolt basic parameters.

Parameter	Numerical Value	Parameter	Numerical Value
Thread center diameter $d_{2}$	7.2 mm	Stress cross-sectional area $A_{S}$	39.167 mm^2^
Diameter of the bolt hole $d_{0}$	8.8 mm	Equivalent diameter $d_{c}$	7.06 mm
Maximum outer diameter of the nut $d_{1}\approx 1.5d$	13.5 mm	Pitch P	1 mm
tan(α+β)	0.19	Yield strength $\sigma_{s}$	640 MPa

**Table 2:** Main technical indexes of the universal testing machine.

Performance Indicator	Parameter Range	Performance Indicators	Parameter Range
Maximum test force	±60 kN	Dynamic fluctuation	±2%
Maximum dynamic test force	±50 kN	Displacement range	±100 mm
Measuring range of test force	2–100% FS	Test frequency range	0.01–50 Hz
Test force accuracy	±1% FS	Rack strength	3.3 × 10^8^ N/m
Two-column main frame	Hard chrome plated		

**Table 3:** JHBM-4 ring force sensor main indicators.

Performance Indicator	Parameter Range
Range	0–20,000 N
Sensitivity	1.0~2.0 ± 0.1 mv/V
Combined accuracy	0.2% F·S
Operating temperature range	−20~+70 °C
Allowable overload	120% F·S
Excitation voltage	5~12 VDC
Creep	±0.1% F·S/30 min

**Table 4:** Main parameters.

Performance Indicator	Parameter Range	Performance Indicator	Parameter Range
Input method	mV signals, standard variable signals or frequency signal	Baud rate	2400, 4800, 9600, 19,200 bps
Sampling speed	10 times/s, 80 times/s	Power consumption	Less than 5 VA
Precision	±0.02% F·S	Operating temperature	−20~50 °C
Communication interface	Standard serial RS-232/485 interfaces	Power supply	220 VAC/24 VDC

**Table 5:** Calculation of pre-loading slope.

Equation	y=a+b⋅x			
Intercept	149.4 ± 319.5	−14.6 ± 576.4	496.6 ± 380.2	−61.5 ± 366.0
Slope	512.3 ± 17.3	509.3 ± 31.3	505.9 ± 20.7	479.2 ± 20.0
Pearson’ s r	0.99829	0.99436	0.99749	0.99739
R-squared (COD)	0.99658	0.98876	0.99499	0.99478
Adjusted R-squared	0.99543	0.98501	0.99332	0.99304

**Table 6:** Fitting accuracy and parameters.

Experimental Category	Fitting Accuracy			Parameter Count		
	Allometric Model Function	Nine-Stage Polynomial Function	Gaussian Function	Allometric Model Function	Nine-Stage Polynomial Function	Gaussian Function
8.8 external hexagonal bolt	0.99794	0.99477	0.99818	2	10	15
8.8 internal hexagonal bolt	0.99919	0.99346	0.99876	2	10	15
12.9 external hexagonal bolt	0.99562	0.98914	0.99814	2	10	15
12.9 internal hexagonal bolt	0.98687	0.98566	0.99917	2	10	15

**Table 7:** Pre-loading decay of different bolts.

Bolt	Torque (Nm)	Initial Pre-Loading (N)	Pre-Loading after Test (N)	Torque Coefficient	Pre-Loading Decay (%)
8.8 external hexagon bolt	22.17	11,567	1402	0.2396	87.9%
8.8 internal hexagon bolt	22.15	10,662	2622	0.2597	75.4%
12.9 external hexagon bolt	22.05	11,083	3803	0.2487	65.7%
12.9 internal hexagon bolt	22.0	10,661	3913	0.2579	63.3%

**Table 8:** Specific attenuation of the bolt.

Displacement	1st	10th	50th	100th
0.25 mm	17.1%	20.5%	21.8%	22.0%
0.5 mm	36.2%	53.5%	65.6%	87.4%
1.0 mm	36.8%	57.1%	87.9%	93.6%
1.5 mm	49.6%	69.8%	92.1%	99.6%
2.0 mm	50.2%	82.7%	99.3%	

**Table 9:** Specific decay of bolts.

Torque	1st	10th	50th	100th
4 Nm	16.2%	54.7%	82.3%	96.3%
10 Nm	36.2%	55.2%	64.8%	69.1%
16 Nm	35.9%	52.8%	75.8%	81.3%
22 Nm	36.8%	57.1%	87.9%	93.6%
28 Nm	38.3%	53.5%	68.3%	76.6%

## Data Availability

Data sharing does not apply to this article.
